# Tempest in a Tea Pot: How did the Public Conversation on Genetically Modified Crops Drift so far from the Facts?

**DOI:** 10.1007/s13181-014-0402-7

**Published:** 2014-05-06

**Authors:** Daniel A. Goldstein

**Affiliations:** Regulatory Affairs, Monsanto Company, Monsanto, Mail Zone C3ND, 800 N.Blvd. St. Louis, Lindbergh, MO 63167 USA

**Keywords:** GM crops, GMO, Plant biotechnology, Food safety, Environmental impact

## Abstract

The debate over genetically modified (GM) crops has raged in Europe since 1996, but had barely risen above a whisper in the USA until recent labeling debates raised public attention. This article will explain GM crops and traits discuss safety assessment provide a view on safety from authoritative organizations discuss selected issues of current debate, and provide the author’s perspective as to why the public debate has drifted so far from scientific reality. The economic and environmental benefits of GM crops are beyond scope, but references are provided. GM food and feed undergo comprehensive assessments using recognized approaches to assure they are as safe as the conventional congener. Issues of food safety and nutrition, unrelated to the GM process, may arise when GM foods display novel components or composition. Unanticipated genetic effects in GM crops appear to be limited in contrast to existing variations among conventional varieties resulting from breeding, mutation, and natural mobile genetic elements. Allergenic potential is assessed when selecting genes for introduction into GM crops and remains a theoretical risk to date. Emerging weed and insect resistance is not unique to GM technology and will require the use of integrated pest management/best practices for pest control. Gene flow from GM crops to wild relatives is limited by existing biological barriers but can at time be a relevant consideration in gene selection and planting practices. Insect-resistant GM crops have significantly reduced use of chemical insecticides and appear to have reduced the incidence of pesticide poisoning in areas where small scale farming and hand application are common. Changes in herbicide patterns are more complex and are evolving over time in response to weed resistance management needs. Recent public debate is driven by a combination of unfounded allegations about the technology and purveyors, pseudoscience, and attempts to apply a strict precautionary principle.

## Public opinion

The public debate over genetically modified (GM) crops has raged in Europe since their introduction in 1996, but had barely risen above a whisper in the USA until the recent California [1] and Washington [2] GM labeling debates raised public attention. If the issue has not entered your world as a medical toxicologist, it may do so soon—either as a debate about GM crops or about the pesticides used with them. This article will explain GM crops and the traits they contain, discuss safety assessment, provide a view on safety from authoritative organizations, discuss selected current matters of legitimate scientific debate such as food safety of compositionally modified crops and the emergence of pest resistance, and provide the author’s perspective as to how the public debate has drifted so very far from scientific reality. The economic and agronomic impacts of GM crops as well as environmental topics not included here have been reviewed elsewhere [3–6].

## Current GM Crops and Safety Assessment

GM crops in use today [3] are largely global commodity crops—maize, soybean, cotton, canola, sugar beet, and alfalfa, alongside a number of nutritionally improved oils such as omega-3 (stearidonic acid, 18:3, n-3) containing soybean oil. Direct human consumption of GM crops is generally limited to sweet corn, papaya, and squash and to consumption of processed fractions (oil, protein, sugar, etc.) derived from GM crops. New genetic material in GM crops is incorporated into the plant’s primary genome and in most cases is transcribed to mRNA and subsequently translated to an effector protein. However, viral resistance and some nutritional traits are the result of RNA-mediated gene regulation, with no novel protein production. DNA and RNA are normal dietary components and present no recognized hazard [7].

Primary GM traits include herbicide tolerance, insect resistance, viral resistance, and nutritional enhancements. Herbicide tolerance facilitates the use of herbicides over GM crops (such as maize, soybean, canola, cotton, and sugar beet), allowing the crop to remain uninjured while weeds are controlled. Tolerance to glyphosate is the leading commercial herbicide-tolerant trait. Glyphosate inhibits the EPSPS (enyol-pyruvate-shikimate-3-phosphate synthase) [8], the first step in the synthesis of aromatic amino acids essential for plant growth and survival. Glyphosate tolerance is mediated by a bacterial EPSPS gene unaffected by glyphosate [8]. Glufosinate tolerance results from the introduction of an acetyltransferase which inactivates the herbicide [8]. Crops tolerant to other herbicides are commercially available (glufosinate, 2,4-D) or under development and undergoing regulatory review (dicamba) [8].

Insect resistance is available in maize, cotton, soybean, and brinjal (eggplant) [8]. Resistance is conferred by a variety of *Cry* (crystal) or *VIP* (vegetative) insecticidal proteins (Cry1Ac, Cry3Bb, Vip3A, etc.) derived from multiple strains of *Bacillus thuringiensis* (Bt) [8]. Bt is a naturally occurring organism used as an insecticide in conventional and organic agriculture for over 50 years. Viral resistance is RNA-mediated (RNAi directed at viral capsid proteins, mimicking the natural plant-defense mechanism) [8]. Altered nutrient composition can be created via RNA-induced gene suppression or by introduction of enzymes conducting desired biochemical reactions [8].

In the USA, GM crops are conjointly regulated by the FDA (food and feed safety), USDA (plant pest risks), and EPA (pesticidal products) under an established inter-agency agreement [9]. There is no specific US Federal legislation regarding GM food safety. All food must be safe (no unreasonable risk of harm under anticipated conditions of use), and GM crops, foods, and ingredients must be as safe as their conventional counterparts. Thus, FDA has the authority to regulate GM food and feed and always has jurisdiction to prevent the sale of any foods/feed it deems to be adulterated. While there is a theoretical pathway to market (self-GRAS determination) which could bypass FDA review, every commercial GM crop has been voluntarily submitted by industry for an FDA premarket safety assessment. The fact that safety assessment of GM crops is mandatory in most jurisdictions outside of the USA, often contingent on prior US approval, assures that pre-market assessment will remain the de facto standard approach to US regulation.

No whole food category in history has been as thoroughly examined as GM crops—only chemical food additives receive greater scrutiny. Pre-market food and feed safety assessment is based on internationally recognized approaches and must demonstrate that GM crops are as safe as their conventional counterparts for food and feed use [10–12] and present no unacceptable risk to the environment [13]. The process begins with a comparative assessment to identify similarities (referred to as substantial equivalence) and differences between the newly developed GM crop and a conventional counterpart with a long history of safe use. Any actual or suspected differences then become the focus of the food, feed, and environmental safety assessment. The assessment [10, 11] begins with careful selection of gene source to avoid allergenic and potentially toxic sources. Food and feed assessment generally focuses on safety of the introduced protein. Bioinformatic (DNA and protein sequence) analysis assures lack of homology to allergens or toxins, and heat stability and digestibility analyses ensure a lack of digestive stability. Acute protein toxicity studies as well as 28- or 90-day whole crop studies are routinely performed in rodents, and livestock studies provide additional assurance of nutritional performance. The crop is subjected to detailed compositional analysis, including known toxins and anti-nutrient factors, “proximate analysis” (total protein, carbohydrate, ash, mineral content, etc.), and analysis of fatty acids, amino acids, vitamins, and minerals—all to assure that the composition of the GM crop falls well within the range of expected values for the conventional crop.

For GM crops with altered or novel compositional aspects (e.g., nutritionally modified oils), a comprehensive safety assessment is undertaken for any changes made, considering both individual health and population nutritional impacts. Unanticipated genetic effects can occur with any breeding technique, and the absence of relevant unanticipated effects can be demonstrated by studies to assure proper gene insertion, composition, and agronomic performance. Fifteen years of studies demonstrate considerably more variability among conventional crops due to genetics and environment than results from transgene insertion in a particular variety; recent genomics studies demonstrate that gene insertion produces minor perturbations of overall gene function compared to dramatic differences in expression across crop varieties, locations, and growing conditions (see below).

Despite this scrutiny, concern is often expressed that no long-term studies with GM crops have been performed. While this is not correct [14], it is also important to recognize that DNA, RNA, and protein are normal dietary components. There are no examples of dietary DNA, RNA, or digestible protein having carcinogenic or reproductive toxicity, and long-term testing to detect these outcomes is neither necessary nor informative. Acute toxicity testing is done using sufficiently high dose (up to 2,000-mg protein/kg body weight) to ensure adequate margins of safety (thousands of times greater than intake). To keep this in perspective, recall that there are tens of thousands of proteins in maize alone—hundreds of thousands in a normal diet—none of which have ever been subjected to long-term testing.

## Legitimate Scientific Debate

Before turning to the increasingly loud public debate on GM crops, it is worth pausing to identify some areas for which legitimate scientific concerns have been expressed. Few of these issues revolve around human health for precisely the reasons discussed above—currently implemented GM crops introduce DNA, RNA, and digestible proteins with a history of safe use into the diet following extensive regulatory review, and are thus unlikely to have associated safety concerns. The topics below are currently in public debate and thus of possible interest to readers. Given available space, this is not a comprehensive list and is not intended to suggest that other issues necessarily lack in scientific merit.

Food-safety-related issues may arise in the context of GM crops with significant alterations in nutrient composition or with novel dietary components. These issues have little to do with GM technology as they relate primarily to impacts on overall dietary consumption patterns and the safety of specific food components. Appropriate safety assessment is nonetheless warranted. For example, the introduction of high-oleic, low-linolenic soybean oil for use in fast-food frying applications can reduce saturated fat intake [15]. No new dietary component is introduced in this product, which results from RNA-mediated gene regulation and conventional breeding and which contains no novel protein [15]. On first blush, one might think no safety issues arise. However, it is reasonable to ask whether widespread use would result in inadequate intake of essential fatty acids in humans, as the oil has reduced levels of linoleic and alpha-linolenic acids. This question was addressed with detailed market modeling to establish no significant effect on essential fatty acid intake [15].

Similarly, the introduction of omega-3 fatty acid containing soybeans enhanced with stearidonic acid (SDA) [16] raises questions about the safety of SDA as a food component. SDA is an 18-carbon fatty acid with four double bonds (18-4, n-3). Conversion of alpha-linolenic acid (ALA; 18-3, n-3) to SDA is the first and rate-limiting step in conversion of ALA to the long-chain fatty acid, eicosapentaenoic acid (EPA) found in fish oil [16, 17] and is expected to provide heart health benefits as a result of conversion to EPA [17]. As such, it is a normal metabolic intermediate in humans. However, only traces of SDA can be found in humans and very little SDA occurs in the human diet. Consequently, SDA soybean oil underwent studies, including a 28-day sub-chronic rat toxicity study with 90-day reproductive and developmental toxicity [18], to establish safe levels of intake in rodents; and SDA soybean oil was subjected to GRAS determination with formal FDA review.

Unanticipated genetic effects of GM technology have been alleged to raise food safety issues. It has long been recognized that conventional technologies (which include wide or forced crosses, plant embryo rescue, and chemical or radiation induced mutagenesis) can also result in unanticipated phenomenon and that the risk of GM technologies falls within the range of risks entailed with conventional methods [19]. In the last several years, genomic technology has documented that the GM process itself produces only small changes in overall gene expression and proteomics in the transformed plant when compared to the large degree of variation introduced by natural genomic instability [20] and by conventional breeding processes and environmental effects [20–27].

Food allergy has also been raised as a potential issue in GM crops. GM crops are, of course, as allergenic as conventional crops as no allergenic components have been removed. As of this writing, there has been no documented occurrence of allergy to an inserted GM protein. Approaches to allergenicity assessment in GM crops have been reviewed elsewhere [28]. By way of summary, when selecting proteins for use in GM crops, we avoid known allergenic sources such as tree nuts; using bioinformatic approaches, we avoid known food allergens as well as proteins having sequence similarity (eight or more amino acids) to known food allergens; and proteins are screened for heat stability and poor digestibility, two characteristics often found in allergenic proteins. Food allergy reduction is theoretically feasible using GM technology [29], but has not yet been developed commercially. It is the author’s view that in the unlikely event that a major food allergen is engineered into a GM crop, it would be removed from commercial use; an option we do not have with most existing, naturally occurring allergens. Thus, the GM allergenicity debate appears to place undue focus on a theoretical and remediable risk while major allergenic foods remain unrestricted (albeit labeled) in the market place.

A complete discussion of the environmental aspects of GM crops is beyond the scope of this paper, but a few recent issues are worthy of note. Given that medical toxicologists are likely less familiar with agronomic and environmental issues, references provided in this section are more introductory in nature.

Agronomically important insect resistance to Bt toxins has emerged as a practical issue in corn rootworm management using single Bt genes for insect control in the upper Midwestern USA [30] and in pink bollworm in India [31], and evidence of field selection for resistance (without clear emergence of heritably resistant populations) has been identified in other species in specific regions [32]. While single traits remain an effective management tool in most locations, the incidence of resistance highlights the need for broader management strategies, commonly called integrated pest management (IPM) or best management practices (BMP). IPM/BMP takes advantage of agricultural interventions (for example, crop rotation) in conjunction with conventional pesticides and multi-trait GM crops with multiple modes of action to limit the emergence of resistant strains, while monitoring for and aggressively managing local resistance where and when it occurs (see, for example, US EPA [33]). This approach approximates the approach we take for management of microbial resistance in hospital environments—altering the environment to reduce effective transmission while simultaneously employing high doses and multiple modes of action to limit resistance emergence and isolating and managing outbreaks of resistant strains.

Weed management is not just a cosmetic issue for farmers—weed competition for resources in the field results in major yield loss if management issues are not properly addressed [34]. Weed resistance to glyphosate, widely used in GM crops, has become an issue with selected weed types and in particular locations over the past decade [32, 35]. This is, as with the food safety issues, less a matter of GM technology itself and more a matter of chemical use patterns. The proliferation of glyphosate tolerant crops has increased the intensity of glyphosate use and has indirectly contributed to this issue [35]. However, herbicide resistance has arisen spontaneously to essentially all classes of herbicide chemistry long before the existence of GM crops [36]. Management techniques [35–37] closely parallel those used in insect resistance: employ multiple chemistries with independent modes of action and apply maximal permitted rates to limit emergence of resistance, employ agronomic practices (mechanical tillage, spot herbicide application, hand weeding) to limit propagation of resistant lines, and monitor for and aggressively manage resistance when and where it occurs. This, of course, has ramifications for overall patterns of herbicide use as discussed below.

Another topic of debate is the possibility of gene flow [38, 39]. Gene flow is the transfer of genetic material (genes) from one population to another [40] and can occur, depending on the plant species, by the relocation of asexual propagules (e.g., tubers) or seed outside of cultivation to produce wild populations or alternatively by sexual reproduction (pollination) [41]. Gene flow is a natural occurrence [42] and is not an inherently adverse phenomenon [40]. Gene flow from GM crops has not lead to any recognized health or environmental problems [40].

Gene flow from crop plants began approximately 10,000 years ago with the domestication of crops [39, 41], during which wild plants were selected for several traits termed the “domestication syndrome” [43]. Some domestication traits limit gene flow. For example, increased seed retention (reduced “shattering”) in crops increases harvest yields and decreases seed-mediated gene flow [40]. Modern cultivated varieties of maize do not occur outside of cultivation and thus, maize is not considered weedy or invasive [41]. However, other crop species have domesticated wild and weedy forms as is the case for sugar beet in Europe [41]. Although most crops have limited seed dispersal mechanisms, seed-mediated gene flow can occur by seed loss during transportation, as occurs with canola (oilseed rape). Dispersed canola populations typically occur in human-disturbed habitats such as roadsides and railways, but do not persist without continual replenishment of the seed bank by human activities because canola does not effectively compete with perennial vegetation in the absence of ongoing disturbances [44, 45]. No differences in soil seed persistence between transgenic herbicide-tolerant and conventional canola have been demonstrated [46–50].

Pollen-mediated gene flow from GM to non-GM crops or to related species has also received significant attention [32]. Pollen may move from GM to non-GM crops in close proximity, with the amount of actual gene flow dependant on distance and reproductive biology. While the organic industry has decided not to use GM seed and some growers are concerned pollen-mediated gene flow, this can be managed successfully to enable coexistence with cooperation among adjacent growers [41].

Pollen-mediated gene flow to non-crop species has been extensively reviewed [41]. Gene flow from crops to weeds has occurred historically and has been implicated in the evolution of enhanced weediness for some species in some geographic regions [51] but did not involve GM crops. For hybridization to occur, related genetically compatible species must be present in the environment. Thus, pollen-mediated gene flow from maize or soybean to related species is not a concern in the USA, because there are no cross compatible relatives [32]. Wild relatives may exist in areas of crop origin as with, for example, maize in Mexico [41]. Although the presence of a wild relative creates the possibility of gene flow, it does not mean that gene flow will necessarily occur [41], as the crop and a sexually compatible relative must flower at the same time [52] and pollen must germinate, grow, and fertilize the ovule to produce a viable seed. The resulting first generation hybrids must then germinate and either self-pollinate or backcross to the related species multiple times to facilitate introgression—the established and sustained transfer of a gene from one population into another [38, 52]. There are several barriers to successful hybridization and introgression, including sterility and reduced fitness of a hybrid in the environment [53]. As noted above, traits associated with the domestication syndrome may limit hybrid fitness in nature [38]. Further, any fitness change to a crop relative associated with a GM trait will not necessarily result in increased fitness, weediness, or invasiveness (recall that the GM gene does not transfer alone—fully, half of the genetic content of the hybrid derives from the domesticated crop). Changes may be detrimental, and some traits may be neutral (provide no benefit or detriment), as with herbicide tolerance in a habitat without the herbicide application [41]. Thus, each crop and trait combination requires a rigorous environment risk assessment to evaluate both the potential for gene flow to related species and the consequences of gene flow if it were to occur [13]. In some instances, these considerations have, in fact, delayed or limited the deployment of GM crops [32].

Finally, it is worth noting the current pesticide-use-reduction debate. A major advantage of Bt insect control has been a dramatic reduction in the need for conventional small-molecule insecticides over Bt commodity crops—most particularly corn and cotton [5, 32]. This has resulted in a net reduction estimated at 50 million kilograms of predominantly organophosphate insecticides (1996–2011) [5] and is a considerable advantage in areas where small-holder agriculture is the norm and application is often by hand as indicated by reductions in pesticide poisoning events [54, 55].

The use of glyphosate tolerant crops initially produced a significant reduction in the use of non-glyphosate herbicides and in total herbicide use [5]. In addition, the use of glyphosate for weed management has generally resulted in displacement of herbicides with less favorable toxicological and environmental characteristics [32]. The emergence of weed resistance has necessitated the increased use of maximum-rate glyphosate applications and has also increased the necessity for alternative herbicides either in conjunction with crops having a broader spectrum of herbicide tolerance (glyphosate, glufosinate, and 2,4-D with dicamba under development) or independent of GM systems [32]. The choice to use herbicide-tolerant cropping systems provides economic and ecological benefits (including promotion of biodiversity) [56] as a result of conservation tillage and other factors [5] and provides additional options for integrated pest management [5, 32], but annual “pounds on the ground” reductions in herbicide use have clearly eroded with the need for weed resistance management (discussed above) [32].

These environmental issues are, on the one hand, real and quite worthy of discussion and debate. They are, however, extensions of ongoing issues already present in conventional agriculture. While there is a clear need to address these issues effectively, this does not appear to necessitate rejection of GM technology as a whole. Rather, the role of GM technology—and various conventional technologies—in agriculture should continue to evolve in response to an evolving environment and in response to continued technical and scientific developments, genetic, and otherwise [6, 32].

### The current public debate

Authoritative organizations such as the FDA, World Health Organization, AAAS, the Royal Society of Medicine, and the National Academy of Science have affirmed the safety of GM crops [9, 19, 57–60]. So, how did the public conversation on GM crops become so negative? Opponents of GM have used three approaches to drive negative public opinion. The first approach has been to create negative impressions about the developers and purveyors of this technology, particularly Monsanto (a Google image search for Monsanto vs. the other major agrochemical companies will provide insight!). At a recent medical meeting, three issues repeatedly arose in casual conversation: (a) Monsanto’s GM crops produce seeds that are sterile (untrue, as are rumors that we developed or use the “terminator” gene [61]), (b) Monsanto sues farmers for inadvertent wind-blown pollination (untrue—Monsanto has sued a small number of farmers who intentionally violated the GM seed purchase agreement by saving and in some cases selling GM seed [62]), and (c) Farmers in India are committing suicide due to the increased economic burden resulting from GM crop failure (unfounded allegation long refuted by studies performed by the government of India and International Food Policy Research Institute [63]). Unfortunately, these kinds of allegations serve to undermine confidence in GM despite an extensive body of scientific evidence to the contrary.

A second approach is to question the science underlying GM safety, often via misinterpretation of data obtained in inappropriate test systems. One recurrent example is the use of surfactant-containing preparations in cell culture systems, “proving” that glyphosate-surfactant products are endocrine disruptors since they impair hormone production. In fact, these systems demonstrate surfactant-mediated disruption of mitochondria, and the authors have simply chosen hormone production as a politically expedient endpoint for cellular asphyxia [64]. Data from more routine systems can also be misinterpreted. A recent egregious example is the now-withdrawn rodent study by Seralini et al. [65] demonstrating tumors and early death in rats—a study not just published but “launched” with a press event, book publication, and videos in three languages. While the pictures are dramatic, no statistics were performed and the findings were within expectation for Sprague-Dawley rats [66 and associated annexes]. Regulatory agencies around the globe wasted untold hours rebutting and explaining these findings. One can also get traction simply by spinning unfounded theories. For example, Samsel and Seneff [67], neither of whom has any background in the biological sciences, published an article in a physics journal (Entropy) arguing that GM crops and associated pesticides act via “exogenous semiotic entropy” and lead to most of the diseases and conditions associated with a Western diet, including gastrointestinal disorders, obesity, diabetes, heart disease, depression, autism, infertility, cancer, and Alzheimer’s disease. Their arguments were quickly rejected and derided, but not before they gained a large amount of press and provided support to the many unfounded fears of GMO’s.

A third approach is to invoke the “precautionary principle.” As originally proposed, the principle stated that risk assessors and policy makers should take account of uncertainties and employ uncertainty factors when necessary to provide adequate margins of safety [68]. The absolute version employed by some suggests that in the presence of any uncertainty, we should not move forward [69]. This is of course a “black hole”—one can never prove a negative and hence one can never move forward when employing the absolute precautionary principle. The latter ignores both the risks of existing technology and the benefits of innovation and leaves decision making in the hands of anyone who chooses to raise doubts.

GM crops have a more than 20-year track record of being grown and used commercially without a single human illness known to be caused by GM food or feed. Moreover, billions of animals have been fed predominantly GM diets for consecutive generations with no evidence that animal health and productivity were affected. The safety assessment paradigm for GM crops is robust and well established, and the approach has been confirmed by authoritative regulatory agencies and scientific organizations around the globe. These are, by far, the most thoroughly assessed foods and feeds in human history, and the National Academies of Science concluded that risk or unintended effects of GM technologies falls within the range of risks for conventional breeding technologies—which include forced inter-species crosses and radiation-induced mutagenesis [19]. We can move forward with high confidence that GM food and feed are as safe and nutritious as their conventional congeners and perhaps look forward to rationalizing food safety assessment across conventional, GM, and other new breeding technologies to achieve a more focused and resource-efficient safety assessment process.
